# Effect of genetic variation in microRNA binding site in WNT1-inducible signaling pathway protein 1 gene on oral squamous cell carcinoma susceptibility

**DOI:** 10.1371/journal.pone.0176246

**Published:** 2017-04-20

**Authors:** Hon-Kit Lau, Edie-Rosmin Wu, Mu-Kuan Chen, Ming-Ju Hsieh, Shun-Fa Yang, Lyu-Yao Wang, Ying-Erh Chou

**Affiliations:** 1 Institute of Medicine, Chung Shan Medical University, Taichung, Taiwan; 2 Department of Anaesthesiology, Asia University Hospital, Taichung, Taiwan; 3 Division of General Surgery, Department of Surgery, Tungs’ Taichung MetroHarbor Hospital, Taichung, Taiwan; 4 Cancer Research Center, Changhua Christian Hospital, Changhua, Taiwan; 5 Department of Otorhinolaryngology-Head and Neck Surgery, Changhua Christian Hospital, Changhua, Taiwan; 6 Graduate Institute of Biomedical Sciences, China Medical University, Taichung, Taiwan; 7 Department of Medical Research, Chung Shan Medical University Hospital, Taichung, Taiwan; 8 School of Medicine, Chung Shan Medical University, Taichung, Taiwan; Fu Jen Catholic University, TAIWAN

## Abstract

**Background:**

Oral squamous cell carcinoma (OSCC), which is the most common head and neck cancer, accounts for 1%–2% of all human malignancies and is characterized by poor prognosis and reduced survival rates. WNT1-inducible signaling pathway protein 1 (WISP1), a cysteine-rich protein belonging to the Cyr61, CTGF, Nov (CCN) family of matricellular proteins, has many developmental functions and may be involved in carcinogenesis. This study investigated *WISP1* single-nucleotide polymorphisms (SNPs) to elucidate OSCC susceptibility and clinicopathologic characteristics.

**Methodology/Principal findings:**

Real-time polymerase chain reaction was used to analyze 6 SNPs of *WISP1* in 900 OSCC patients and 1200 cancer-free controls. The results showed that *WISP1* rs2929970 polymorphism carriers with at least one G allele were susceptible to OSCC. Moreover, compared with smokers, non-smoker patients with higher frequencies of *WISP1* rs2929970 (AG + GG) variants had a late stage (stages III and IV) and a large tumor size. In addition, OSCC patients who were betel quid chewers and carried *WISP1* rs16893344 (CT + TT) variants had a low risk of lymph node metastasis.

**Conclusion:**

Our results demonstrate that a joint effect of *WISP1* rs2929970 with smoking as well as *WISP1* rs16893344 with betel nut chewing causally contributes to the occurrence of OSCC. *WISP1* polymorphism may serve as a marker or a therapeutic target in OSCC.

## Introduction

Oral squamous cell carcinoma (OSCC), which is the most common head and neck cancer, accounts for 1%–2% of all human malignancies. OSCC accounts for more than 90% of oral malignancies [1]. OSCC is characterized by poor prognosis and reduced survival rates [2]. Despite improved imaging techniques and advances in surgery, chemotherapy, and radiation, the prognosis and mortality of OSCC remain poor, with a 5-year survival rate of approximately 50% [3–6].

The Cyr61, CTGF, Nov (CCN) family is a group of 6 secreted proteins that regulates adhesion and migration or functions as growth factors that modulate cell proliferation and differentiation [7]. WNT1-inducible signaling pathway protein 1 (WISP1) is a cysteine-rich protein belonging to the CCN family of matricellular proteins, and WISP1 has many developmental functions [8, 9]. Increasing evidence suggests that WISP1 is involved in carcinogenesis [10]. Research on colon cancer revealed that high WISP1 expression is associated with apoptosis, invasion, and poor prognosis [11]. In esophageal squamous cell carcinoma, WISP1 has been found to be an independent prognostic factor for poor overall survival and has been confirmed to mediate resistance to radiotherapy [12, 13]. In OSCC, WISP1 expression is regulated by methylation, and WISP1 hypomethylation contributes to lymph node (LN) metastasis [14]. Moreover, a previous study indicated that WISP1 enhances the migration of OSCC cells by increasing intercellular adhesion molecule-1 (ICAM-1) expression through the αvβ3 integrin receptor and apoptosis signal-regulating kinase 1 (ASK1), c-Jun N-terminal kinase (JNK)/p38, and activator protein-1 (AP-1) signal transduction pathways [15]. WISP1 may also promote OSCC angiogenesis through vascular endothelial growth factor (VEGF)-A expression [16]. Thus, WISP1 can act as an oncogene and may be a promising therapeutic target in OSCC.

Single-nucleotide polymorphism (SNP) is the most common genetic variant in DNA expression, and the expression of a specific gene may be affected or regulated by its genetic variations [17–21]. Previous data have demonstrated the possible role of WISP1 SNPs in various cancers or diseases [22–26]. For example, in postmenopausal Japanese women, the rs2929970 SNP in the WISP1 3′-UTR region was suggested to be associated with spinal osteoarthritis [24]. WISP1 rs2929970 was also suggested to be correlated with hypertension in men, and men carrying the G allele of rs2929970 had higher blood pressure [26]. Moreover, WISP1 rs2929973 was associated with lung function in asthmatic children [25]. In lung cancer, a series of WISP1 genetic polymorphisms, such as rs16893344, rs2977530, rs2977537, rs62514004, rs11778573, rs2977536, and rs2977549, were significantly associated with lung cancer susceptibility or the chemotherapy response [22]. However, little is known about the effects of *WISP1* gene variants on the predisposition to OSCC. Therefore, we hypothesized that polymorphisms of the *WISP1* gene may play an essential role in OSCC. In this study, we aimed to investigate the *WISP1* SNPs of rs62514004, rs16893344, rs2977530, rs2977537, rs2929970 and rs2929973, which are located in the promoter or the 3′-UTR region, to elucidate their contribution to OSCC susceptibility and clinicopathologic characteristics.

## Materials and methods

### Subjects and specimen collection

We enrolled 900 male patients with OSCC from Chung Shan Medical University Hospital in Taichung and Changhua Christian Hospital in Changhua, Taiwan, from 2007 to 2016. For the control group, we selected 1200 healthy male individuals with no self-reported history of cancer at any site from Taiwan Biobank. Each subject completed a questionnaire on demographic characteristics, betel quid chewing, tobacco use, and alcohol consumption, and medical histories. The Institutional Review Board of Chung Shan Medical University Hospital approved this study (CSMUH No: CS13214-1), and informed written consent was obtained from each participant.

### Selection of *WISP1* polymorphisms

A total of six SNPs in *WISP1* were selected from the International HapMap Project data for this study. The previous studies have reported the effect of *WISP1* genetic polymorphisms on human cancer susceptibility [22, 23, 27]. Thus, we included the SNP rs62514004, rs2929970 and rs2929973 in the promoter region and 3' untranslated region, respectively. Three SNPs (rs16893344, rs2977530 and rs2977537) which locate in introns of *WISP1* were selected in this study since these SNPs were suggested to be significantly associated with lung cancer susceptibility or chemotherapy response in Chinese population [22].

### DNA extraction and *WISP1* genotyping

Genomic DNA from OSCC group was isolated from peripheral blood using the QIAamp DNA blood mini kit and used as the template for polymerase chain reaction as described previously [28, 29]. Allelic discrimination of the rs62514004, rs16893344, rs2977530, rs2977537, rs2929970 and rs2929973 of *WISP1* gene was assessed with the ABI StepOne Real-Time PCR System (Applied Biosystems, Foster City, CA, USA), and analyzed with SDS vers. 3.0 software using the TaqMan assay.

### Bioinformatics analysis

The stem-loop portion of the miRNA-miRNA duplex structure of pre-miRNAs was identified by miRNA target prediction using the MicroRNA.org resource. Models of the miRNA-target duplex were determined using the RNAhybrid web tool on Bielefeld Bioinformatics Server. Boxplot chart showing the differential expressions of miR-99a-5p in 420 OSCC patients and 43 normal controls, as taken from the Pan-Cancer dataset [30].

### Statistical analysis

The Mann-Whitney U test was used to compare differences in the distribution of age and demographic characteristics between the controls and OSCC patients. The common haplotypes were estimated by PHASE version 2.1. ORs with 95% CIs were estimated using logistic regression models. AORs with 95% CIs were used to assess association between genotype frequencies with OSCC risk and clinical factors. p values less than 0.05 were considered significant. The data were analysed with SPSS 12.0 statistical software (SPSS Inc., Chicago, IL, USA).

## Results

The statistical analysis of demographic characteristics is shown in Table 1. We analyzed the demographic characteristics of sample specimens and observed that 16.6% (199/1200) of controls and 78.6% (707/900) of OSCC patients had the habit of betel quid chewing. Moreover, 53.0% (636/1200) of controls and 88.9% (800/900) of OSCC patients were smokers. In addition, 19.7% (237/1200) of controls and 53.0% (480/900) of OSCC patients consumed alcohol. Significant distributional differences were observed in betel quid chewing (p < 0.001), cigarette smoking (p < 0.001), and alcohol consumption (p < 0.001) between controls and OSCC patients.

**Table 1:** The distributions of demographical characteristics in 1200 controls and 900 male patients with oral cancer.

Variable	Controls (N = 1200)	Patients (N = 900)	p value
Age (yrs)	53.91 ± 10.02	55.08 ± 11.09	
<55	566 (47.2%)	440 (48.9%)	p = 0.434
≥ 55	634 (52.8%)	460 (51.1%)	
Betel quid chewing			
No	1001 (83.4%)	193 (21.4%)	
Yes	199 (16.6%)	707 (78.6%)	p <0.001*
Cigarette smoking			
No	564 (47.0%)	100 (11.1%)	
Yes	636 (53.0%)	800 (88.9%)	p <0.001*
Alcohol drinking			
No	963 (80.3%)	420 (46.7%)	
Yes	237 (19.7%)	480 (53.3%)	p <0.001*
Stage			
I+II		444 (49.3%)	
III+IV		456 (50.7%)	
Tumor T status			
T1+T2		519 (57.7%)	
T3+T4		381 (42.3%)	
Lymph node status			
N0		606 (67.3%)	
N1+N2+N3		294 (32.7%)	
Metastasis			
M0		890 (98.9%)	
M1		10 (1.1%)	
Cell differentiation			
Well differentiated		127 (14.1%)	
Moderately or poorly differentiated		773 (85.9%)	

Mann-Whitney U test was used between healthy controls and patients with oral cancer.

* p value < 0.05 as statistically significant.

The genotype distributions and associations between OSCC and the *WISP1* genetic polymorphisms are shown in Table 2. The *WISP1* rs62514004, rs16893344, rs2977530, rs2977537, rs2929970 and rs2929973 genetic polymorphisms exhibited the highest distribution frequency in both control and OSCC patients homozygous for AA, homozygous for CC, heterozygous for AG, heterozygous for GA, heterozygous for AG and heterozygous for TG, respectively. In these controls, the frequencies of *WISP1* rs62514004, rs16893344, rs2977530, rs2977537, rs2929970 and rs2929973 were in the Hardy-Weinberg equilibrium (p = 0.442, χ2 value: 0.590; p = 0.957, χ2 value: 0.003; p = 0.132, χ2 value: 2.274; p = 0.290, χ2 value: 1.119; p = 0.490, χ2 value: 0.476; and p = 0.314, χ2 value: 1.014, respectively). After adjustment for several variables, no significant differences were observed between OSCC patients and control group in the rs62514004, rs16893344, rs2977530, rs2977537 and rs2929973 polymorphisms of the *WISP1* gene. However, patients with *WISP1* polymorphic rs2929970 GG genotypes exhibited a higher risk of OSCC than the corresponding WT homozygous patients (OR [odds ratios] = 1.463, 95% confidence interval [CI] = 1.045–2.048, p = 0.027). Moreover, a similar result was observed in patients with *WISP1* polymorphic rs2929970 AG + GG genotypes (OR = 1.298, 95% CI = 1.026–1.642, p = 0.030).

**Table 2:** Odds ratio (OR) and 95% confidence interval (CI) of oral cancer associated with *WISP1* genotypic frequencies.

Variable	Controls (N = 1200) n (%)	Patients (N = 900) n (%)	OR (95% CI)	AOR (95% CI)
**rs62514004**				
AA	926 (77.2%)	707 (78.6%)	1.00	1.00
AG	253 (21.1%)	180 (20.0%)	0.932 (0.752–1.155)	0.956 (0.726–1.259)
GG	21 (1.7%)	13 (1.4%)	0.811 (0.403–1.630)	0.682 (0.276–1.687)
AG+GG	274 (22.8%)	193 (21.4%)	0.923 (0.749–1.137)	0.943 (0.714–1.211)
**rs16893344**				
CC	877 (73.1%)	662 (73.6%)	1.00	1.00
CT	298 (24.8%)	222 (24.7%)	0.987 (0.807–1.207)	0.956 (0.737–1.239)
TT	25 (2.1%)	16 (1.8%)	0.848 (0.449–1.601)	0.795 (0.349–1.812)
CT+TT	323 (26.9%)	238 (26.4%)	0.976 (0.803–1.187)	0.943 (0.733–1.214)
**rs2977530**				
AA	337 (28.1%)	263 (29.2%)	1.00	1.00
AG	573 (47.8%)	435 (48.3%)	0.973 (0.793–1.193)	0.840 (0.645–1.093)
GG	290 (24.1%)	202 (22.5%)	0.893 (0.701–1.136)	0.810 (0.592–1.108)
AG+GG	863 (71.9%)	637 (70.8%)	0.946 (0.781–1.145)	0.830 (0.648–1.063)
**rs2977537**				
GG	330 (27.5%)	233 (25.9%)	1.00	1.00
GA	581 (48.4%)	435 (48.3%)	1.060 (0.861–1.307)	1.033 (0.789–1.353)
AA	289 (24.1%)	232 (25.8%)	1.137 (0.894–1.447)	1.191 (0.873–1.625)
GA+AA	870 (72.5%)	667 (74.1%)	1.086 (0.893–1.321)	1.085 (0.842–1.397)
**rs2929970**				
AA	439 (36.6%)	302 (33.6%)	1.00	1.00
AG	583 (48.6%)	443 (49.2%)	1.105 (0.912–1.338)	1.248 (0.973–1.600)
GG	178 (14.8%)	155 (17.2%)	1.266 (0.976–1.642)	1.463 (1.045–2.048)*
AG+GG	761 (63.4%)	598 (66.4%)	1.142 (0.953–1.370)	1.298 (1.026–1.642)*
**rs2929973**				
TT	503 (41.9%)	395 (43.9%)	1.00	1.00
TG	560 (46.7%)	391 (43.4%)	0.889 (0.739–1.069)	0.976 (0.769–1.238)
GG	137 (11.4%)	114 (12.7%)	1.060 (0.800–1.404)	1.133 (0.789–1.627)
TG+GG	697 (58.1%)	505 (56.1%)	0.923 (0.775–1.099)	1.007 (0.804–1.262)

The odds ratio (OR) with their 95% confidence intervals were estimated by logistic regression models.

The adjusted odds ratio (AOR) with their 95% confidence intervals were estimated by multiple logistic regression models after controlling for betel nut chewing, alcohol and tobacco consumption.

The interactive effects between environmental risk factors and the *WISP1* genetic polymorphisms are shown in Table 3. To reduce the possible interference of confounding variables, we used adjusted ORs (AORs) with 95% CIs that were estimated using multiple logistic regression models after controlling for betel nut chewing and alcohol consumption in each comparison. Among 664 non-smokers, those with *WISP1* rs2977530 AG + GG genotypes exhibited a 0.598 fold-lower risk of OSCC (95% CI = 0.364–0.980, p = 0.041) (Table 3) but no difference was observed among 1436 smokers (Table A in S1 File).

**Table 3:** Odds ratio (OR) and 95% confidence interval (CI) of oral cancer associated with *WISP1* genotypic frequencies in non-smoker.

Variable	Controls (N = 564) n (%)	Patients (N = 100) n (%)	OR (95% CI)	AOR (95% CI)
**rs62514004**				
AA	444 (78.7%)	78 (78.0%)	1.00	1.00
AG	110 (19.5%)	22 (22.0%)	1.138 (0.679–1.909)	1.096 (0.610–1.968)
GG	10 (1.8%)	0 (0%)	---	---
AG+GG	120 (21.3%)	22 (22.0%)	1.044 (0.624–1.745)	1.014 (0.567–1.814)
**rs16893344**				
CC	411 (72.9%)	75 (75.0%)	1.00	1.00
CT	141 (25.0%)	24 (24.0%)	0.933 (0.567–1.535)	0.784 (0.441–1.397)
TT	12 (2.1%)	1 (1.0%)	0.457 (0.059–3.564)	0.172 (0.016–1.894)
CT+TT	153 (27.1%)	25 (25.0%)	0.895 (0.549–1.460)	0.720 (0.407–1.273)
**rs2977530**				
AA	165 (29.3%)	41 (41.0%)	1.00	1.00
AG	261 (46.3%)	40 (40.0%)	0.617 (0.383–0.994)*	0.585 (0.340–1.006)
GG	138 (24.4%)	19 (19.0%)	0.554 (0.307–0.999)*	0.623 (0.325–1.197)
AG+GG	399 (70.7%)	59 (59.0%)	0.595 (0.384–0.922)*	0.598 (0.364–0.980)*
**rs2977537**				
GG	153 (27.1%)	27 (27.0%)	1.00	1.00
GA	274 (48.6%)	41 (41.0%)	0.848 (0.502–1.433)	0.879 (0.489–1.578)
AA	137 (24.3%)	32 (32.0%)	1.324 (0.755–2.321)	1.180 (0.622–2.237)
GA+AA	411 (72.9%)	73 (73.0%)	1.006 (0.623–1.625)	0.982 (0.573–1.684)
**rs2929970**				
AA	203 (36.0%)	31 (31.0%)	1.00	1.00
AG	271 (48.0%)	50 (50.0%)	1.208 (0.745–1.960)	1.127 (0.657–1.932)
GG	90 (16.0%)	19 (19.0%)	1.382 (0.742–2.577)	1.120 (0.550–2.284)
AG+GG	361 (64.0%)	69 (69.0%)	1.252 (0.792–1.977)	1.125 (0.675–1.875)
**rs2929973**				
TT	233 (41.3%)	46 (46.0%)	1.00	1.00
TG	263 (46.6%)	43 (43.0%)	0.828 (0.527–1.301)	0.810 (0.487–1.347)
GG	68 (12.1%)	11 (11.0%)	0.819 (0.402–1.668)	0.695 (0.307–1.571)
TG+GG	331 (58.7%)	54 (54.0%)	0.826 (0.539–1.267)	0.785 (0.485–1.272)

The odds ratio (OR) with their 95% confidence intervals were estimated by logistic regression models.

The adjusted odds ratio (AOR) with their 95% confidence intervals were estimated by multiple logistic regression models after controlling for betel nut chewing and alcohol.

We used Haploview software and the PHASE program to analyze the common haplotypes. As shown in Table 4, compared with the reference group G-A-T (*WISP1* rs2977537/rs2929970/rs2929973), carriers with G-G-T or A-G-T had 1.857-fold (95% CI 1.374–2.510) and 2.048-fold (95% CI 1.217–3.448) significantly increased risks of OSCC (Table 4).

**Table 4:** Frequencies of *WISP1* haplotypes in OSCC patients and control subjects.

Haplotype block			Controls	Patients	
rs2977537 G/A	rs2929970 A/G	rs2929973 T/G	n = 2400	n = 1800	OR (95% CI)
G	A	T	852 (35.5%)	599 (33.3%)	1.000 (reference)
A	A	T	604 (25.2%)	435 (24.2%)	1.024 (0.872–1.204)
A	G	G	530 (22.1%)	422 (23.4%)	1.133 (0.960–1.336)
G	G	G	299 (12.5%)	184 (10.2%)	0.875 (0.709–1.081)
G	G	T	85 (3.5%)	111 (6.2%)	1.857 (1.374–2.510)^a^
A	G	T	25 (1.0%)	36 (2.0%)	2.048 (1.217–3.448)^b^
G	A	G	5 (0.2%)	7 (0.4%)	1.991 (0.629–6.304)
A	A	G	0 (0.0%)	6 (0.3%)	-

^a^ p< 0.001

^b^ p = 0.007

To clarify the role of the *WISP1* genetic polymorphisms in OSCC clinicopathologic statuses, such as clinical stage, tumor size, LN metastasis, distant metastasis, and cell differentiation, the distribution frequency of clinical statuses and *WISP1* genotype frequencies in OSCC patients were estimated. The rs62514004, rs2977530, rs2977537 and rs2929973 genetic polymorphisms showed no significant association with the clinicopathologic statuses. However, among 707 OSCC patients who were betel quid chewers, those carrying the polymorphic rs16893344 gene had a lower risk of LN metastasis (OR = 0.674, 95% CI = 0.465–0.979, p = 0.038) than those carrying the rs16893344 WT gene, but no difference was observed in clinical stage, tumor size, distant metastasis, or cell differentiation (Table 5). However, no significant differences were observed among 193 non-betel quid chewers (Table B in S1 File). Among 100 non-smoker OSCC patients, those carrying the polymorphic rs2929970 gene had a higher risk of late-stage (OR = 2.428, 95% CI = 0.998–5.909, p = 0.048) and a larger tumor size (OR = 2.965, 95% CI = 1.129–7.789, p = 0.024) than those carrying the rs2929970 WT gene, but no difference was observed in LN metastasis and cell differentiation (Table 6). Moreover, Among 800 smoker OSCC patients, no difference was observed in stage, tumor size, LN metastasis, distant metastasis and cell differentiation (Table C in S1 File).

**Table 5:** Clinical statuses and *WISP1* rs16893344 genotype frequencies in oral cancer among 707 betel quid chewers.

Variable	*WISP1* rs16893344 (betel quid chewers)			
	CC (n = 518) n (%)	CT+TT (n = 189) n (%)	OR (95% CI)	p value
**Clinical Stage**				
Stage I/II	251 (48.5%)	98 (51.9%)	1.00	p = 0.424
Stage III/IV	267 (51.5%)	91 (48.1%)	0.873 (0.625–1.218)	
**Tumor size**				
≤T2	292 (56.4%)	105 (55.6%)	1.00	p = 0.847
> T2	226 (43.6%)	84 (44.4%)	1.034 (0.739–1.445)	
**Lymph node metastasis**				
No	341 (65.8%)	140 (74.1%)	1.00	p = 0.038*
Yes	177 (34.2%)	49 (25.9%)	0.674 (0.465–0.979)	
**Distant metastasis**				
No	512 (98.8%)	187 (98.9%)	1.00	p = 0.911
Yes	6 (1.2%)	2 (1.1%)	0.913 (0.183–4.561)	
**Cell differentiation**				
well	73 (14.1%)	32 (16.9%)	1.00	p = 0.348
Moderate/poor	445 (85.9%)	157 (83.1%)	0.805 (0.511–1.267)	

* p<0.05

**Table 6:** Clinical statuses and *WISP1* rs2929970 genotype frequencies in oral cancer among 100 non-smoker.

Variable	*WISP1* rs2929970			
	AA (n = 31) n (%)	AG+GG (n = 69) n (%)	OR (95% CI)	p value
**Clinical Stage**				
Stage I/II	21 (67.7%)	32 (46.4%)	1.00	p = 0.048*
Stage III/IV	10 (32.3%)	37 (53.6%)	2.428 (0.998–5.909)	
**Tumor size**				
≤T2	24 (77.4%)	37 (53.6%)	1.00	p = 0.024*
> T2	7 (22.6%)	32 (46.4%)	2.965 (1.129–7.789)	
**Lymph node metastasis**				
No	21 (67.7%)	44 (63.8%)	1.00	p = 0.700
Yes	10 (32.3%)	25 (36.2%)	1.193 (0.486–2.932)	
**Cell differentiation**				
well	4 (12.9%)	5 (7.2%)	1.00	p = 0.361
Moderate/poor	27 (87.1%)	64 (92.8%)	1.896 (0.473–7.610)	

* p<0.05

**Fig 1:**
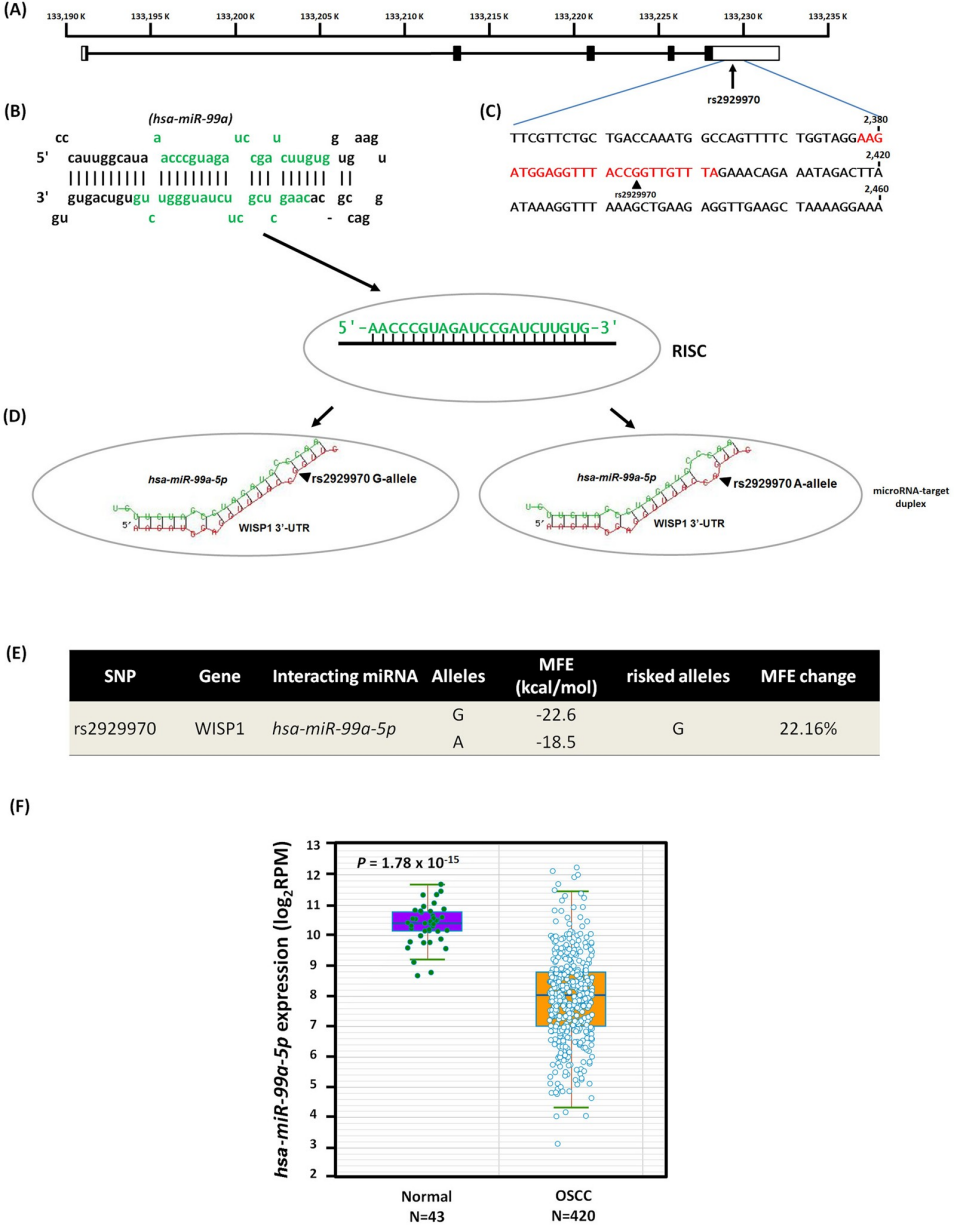
Binding site polymorphism from SNP rs2929970 [G/A] in human WISP1 3’-UTR mRNA with microRNA hsa-miR-99a-5p to decrease oral cancer susceptibility among Taiwan HNSCC population. (A) Exons of WISP1 are shown by the filled boxes from the chromosome positions (chr.13, reference genome GRCh37.p13). (B) The stem-loop portion of miRNA-miRNA duplex structure on pre-miRNAs (hsa-miR-99a; miRBase ID: MI0003190) was identified by microRNA target prediction on MicroRNA.org resource. The hsa-miR-99a-5p sequence marked by green fonts. (C) Sequence of the human WISP1 3’-UTR region and number shown the positions of mRNA (NM_003882). Predicted hsa-miR-99a-5p binding site with SNP rs2929970 was highlighted by color red fonts. (D) The models of microRNA-target duplex were determined using the RNAhybrid web tool on the Bielefeld Bioinformatics Server. RISC, RNA-induced silencing complex, arrows indicate the locus of rs2929970. (E) The SNP rs2929970 A-allele reduces the free binding energy (MFE, minimum free energy; change: 22.16%). (F) Boxplot chart counting the differential expressions of microRNA hsa-miR-99a-5p in the 420 OSCC patients and 43 normal from Pan-Cancer dataset.

As shown in Fig 1A, the 3′-untranslated region (3′-UTR) of the *WISP1* gene is 5.0 kb long and might be among the region’s most sensitive to microRNA (miRNA) epigenetic regulation (Fig 1A). The miRNA *hsa-miR-99a* (miRBase ID: MI0003190, Fig 1B) shares binding site complementarily with rs2929970 in the 3′-UTR region (Fig 1C and 1D). In addition, compared with the [A]-allele, the OSCC-associated risk [G]-allele creates a slight kink in the *WISP1* mRNA structure, which results in a less negative free energy state and less stable hybridization [MFE (minimum free energy) changes: 22.16%, from −22.6 to −18.5 kcal/mol] (Fig 1E). Furthermore, starBase analysis revealed a significant difference in *hsa-miR-99a* expression in 420 OSCC patients and 43 subjects from the Pan-Cancer data set [30] (Fig 1F) (p = 1.78 × 10^−15^).

## Discussion

In this study, we revealed the correlations between *WISP1* SNPs and OSCC. Previous studies have indicated the correlations of risk factors such as alcohol consumption, betel quid chewing, and smoking with oral cancer carcinogenesis [31–33]. In our study, we confirmed that betel quid chewing, cigarette smoking, and alcohol consumption are associated with OSCC (Table 1). However, the associations of these risk factors with WISP1 regulation have yet to be extensively investigated. Smoking is one of the crucial risk factors for OSCC [31, 33]. In a pilot study, whole genome expression profiling of Indian patients with tobacco chewing-associated oral cancers implicated that WISP1 is one of the representative apoptosis-related deregulated genes in oral cancer [34]. Chen et al. revealed that WISP1 was overexpressed in non–small cell lung carcinoma (NSCLC) samples compared with their normal lung tissue counterparts, implicating that WISP1 might act as an oncoprotein in NSCLC [10]. However, WISP1 expression was not associated with clinical parameters such as family history, metastasis, smoking history, tuberculosis, gender, tumor type, and tumor size in NSCLC individuals [10]. *WISP1* mRNA expression levels were higher in both lung and head and neck tumor tissues compared with their normal tissue counterparts [35], and smoking history was inconsistent. Therefore, it can be assumed that smoking has a limited ability to induce WISP1 overexpression.

Recent studies have suggested the vital role of WISP1 in cancer [35–37]. However, the *WISP1* SNPs contributing to cancer progression have yet to be extensively investigated. In the present study, we found that the *WISP1* SNP of rs2929970 was associated with OSCC risk (Table 2); rs2929970 is located in the 3′ untranslated region within a region of splicing variation [38]. Our present study confirmed the association of the clinically examined rs2929970 in the *WISP1* 3′-UTR region with OSCC risk; this association is most likely attributed to a putative hsa-miRNA-99a binding site (Fig 1). This finding suggests that the SNP corresponding to the RNA bulge region may affect the binding strength of specific miRNA/target duplexes, resulting in low minimum free energy and modulating mRNA stability.

A previous study indicated that the rs2929970 SNP may be associated with spinal osteoarthritis in postmenopausal Japanese women [24]. Yamada et al. [26] indicated that *WISP1* rs2929970 was associated with hypertension in men carrying the G allele, and the men carrying this polymorphism had higher blood pressure. These results demonstrate the different effects of *WISP1* rs2929970 expression in different diseases. Such inconsistent results for *WISP1* rs2929970 expression may be due to different ethnicities or diseases; therefore, different genotype distributions may be observed. Although the detailed mechanism of *WISP1* rs2929970 remains unclear, the *WISP1* rs2929970 polymorphism certainly plays a role in cancers or diseases.

In the present study, we determined that nonsmoker controls and OSCC patients with *WISP1* polymorphic rs2977530 AG + GG genotypes had a low risk of OSCC (AOR = 0.598, Table 3). Chen et al. indicated that lung cancer patients carrying the A alleles of *WISP1* rs2977530 polymorphisms may have an increased risk of lung cancer [22]. However, in that study, the smoking status of controls was not adjusted [22]. Consistent with this result, our data reveal that the G alleles of *WISP1* rs2977530 were associated with a low risk of OSCC. We also analyzed the *WISP1* SNP of rs1689334 and showed that 707 OSCC betel quid chewers carrying *WISP1* rs16893344 SNP CT + TT genotypes exhibited a low risk of LN metastasis (OR = 0.674, p = 0.038, Table 5). However, Chen et al. [22] indicated that lung cancer patients carrying the T allele of the *WISP1* rs16893344 polymorphism may have an increased risk of lung cancer. Tao et al. also showed that *WISP1* rs16893344 C > T polymorphisms significantly increased myocardial infarction risk [39]. These results demonstrate that the various *WISP1* SNPs may be expressed in different cancers and diseases. Moreover, a previous study suggested that WISP1 expression is regulated by methylation, and WISP1 hypomethylation contributes to LN metastasis in OSCC [14]. The WISP1 expression is correlated with the DNA methylation of its promoter, and reduced methylation levels are correlated with increased WISP1 expression [14]. Therefore, although the interaction of betel quid chewing with WISP1 expression and the functions of *WISP1* rs16893344 have not been extensively investigated, the *WISP1* SNP of rs16893344 may contribute to changes that influence WISP1 gene transcription.

We analyzed the correlations of *WISP1* SNP expression with the clinical statues of OSCC patients. We observed that among 100 nonsmoker OSCC patients, those carrying *WISP1* rs2929970 AG + GG genotypes had later stage OSCC and a larger tumor size (Table 6). Because smoking is a well-known risk factor for OSCC [31, 33], this result in nonsmoker OSCC patients implicated the pivotal role of the *WISP1* SNP of rs2929970 in cancer progression and WISP1 regulation. A previous study showed that WISP1 binds to αvβ3 integrin and causes the activation of the ASK1, JNK/p38, and AP-1 pathways, which upregulate ICAM-1 expression and promote the migration of human OSCC cells [15], and tumor-secreted WISP1 promotes angiogenesis through VEGF-A expression and increased angiogenesis-related tumor growth [16]. Although the mechanism and regulation of *WISP1* SNPs in cancer progression have not been extensively investigated, the *WISP1* SNPs of rs16893344 and rs2929970 might be involved in WISP1 regulation through WISP1-induced ICAM-1 upregulation and VEGF-A expression. The *WISP1* SNPs of rs16893344 and rs2929970 may interfere with or enhance the binding activity of WISP1 to αvβ3 integrin, triggering the regulation of the ASK1, JNK/p38, and AP-1 pathways and VEGF-A expression, leading to a more favorable or poorer prognosis. Moreover, Mercer et al. also reports that, in a rat model of alcohol-induced liver disease, chronic alcohol consumption can significant upregulation in WISP1 expression [40]. Approximately half of OSCC patients (53.3%) in our study consumed alcohol (Table 1), these OSCC patients may exhibit a higher level of serum WISP1, and alcohol consumption may exert a synergistic effect on WISP1 upregulation. However, the *WISP1* SNPs contributing to cancer progression and WISP1 regulation require further investigation to elucidate their detailed mechanisms.

In conclusion, *WISP1* SNPs are correlated with OSCC. The *WISP1* SNP of rs2929970 is associated with OSCC susceptibility, and rs2929970 A/G polymorphisms may be correlated with a worse prognosis of OSCC, such as later stage OSCC or larger tumor size. *WISP1* rs2929970 may serve as a marker or a therapeutic target in OSCC.

## Supporting information

S1 File Table A. Odds ratio (OR) and 95% confidence interval (CI) of oral cancer associated with WISP1 genotypic frequencies in smoker. Table B. Clinical statuses and WISP1 rs16893344 genotype frequencies in oral cancer among 193 non-betel quid chewers. Table C. Clinical statuses and WISP1 rs2929970 genotype frequencies in oral cancer among 800 smoker.
(DOCX)
