# Supplementary material for: Effect of genetic variation in microRNA binding site in WNT1-inducible signaling pathway protein 1 gene on oral squamous cell carcinoma susceptibility
Source: PLoS One. 2017 Apr 20;12(4):e0176246. doi: 10.1371/journal.pone.0176246 (PMC5398667; doi:10.1371/journal.pone.0176246)
Supplement: S1 File — Table A. Odds ratio (OR) and 95% confidence interval (CI) of oral cancer associated with WISP1 genotypic frequencies in smoker. Table B. Clinical statuses and WISP1 rs16893344 genotype frequencies in oral cancer among 193 non-betel quid chewers. Table C. Clinical statuses and WISP1 rs2929970 genotype frequencies in oral cancer among 800 smoker. (DOCX) [file pone.0176246.s001.docx]

**Table A.** Odds ratio (OR) and 95% confidence interval (CI) of oral cancer associated with *WISP1* genotypic frequencies in smoker.

| Variable | Controls (N=636) n (%) | Patients (N=800) n (%) | OR (95% CI) | p value |
| --- | --- | --- | --- | --- |
| **rs62514004** |  |  |  |  |
| AA | 482 (75.8%) | 629 (78.6%) | 1.00 |  |
| AG | 143 (22.5%) | 158 (19.8%) | 0.847 (0.656-1.093) | p=0.202 |
| GG | 11 (1.7%) | 13 (1.6%) | 0.906 (0.402-2.039) | p=0.811 |
| AG+GG | 154 (24.2%) | 171 (21.4%) | 0.851 (0.664-1.090) | p=0.202 |
| **rs16893344** |  |  |  |  |
| CC | 466 (73.3%) | 587 (73.4%) | 1.00 |  |
| CT | 157 (24.7%) | 198 (24.8%) | 1.001 (0.786-1.276) | p=0.992 |
| TT | 13 (2.0%) | 15 (1.8%) | 0.916 (0.432-1.944) | p=0.819 |
| CT+TT | 170 (26.7%) | 213 (26.6%) | 0.995 (0.786-1.259) | p=0.965 |
| **rs2977530** |  |  |  |  |
| AA | 172 (27.0%) | 222 (27.8%) | 1.00 |  |
| AG | 312 (49.1%) | 395 (49.4%) | 0.981 (0.765-1.257) | p=0.879 |
| GG | 152 (23.9%) | 183 (22.9%) | 0.933 (0.696-1.250) | p=0.642 |
| AG+GG | 464 (73.0%) | 578 (72.3%) | 0.965 (0.764-1.219) | p=0.766 |
| **rs2977537** |  |  |  |  |
| GG | 177 (27.8%) | 206 (25.8%) | 1.00 |  |
| GA | 307 (48.3%) | 394 (49.3%) | 1.103 (0.859-1.416) | p=0.444 |
| AA | 152 (23.9%) | 200 (24.9%) | 1.131 (0.845-1.513) | p=0.409 |
| GA+AA | 459 (72.2%) | 594 (74.3%) | 1.112 (0.879-1.406) | p=0.376 |
| **rs2929970** |  |  |  |  |
| AA | 236 (37.1%) | 271 (33.9%) | 1.00 |  |
| AG | 312 (49.1%) | 393 (49.1%) | 1.097 (0.872-1.380) | p=0.429 |
| GG | 88 (13.8%) | 136 (17.0%) | 1.346 (0.977-1.853) | p=0.069 |
| AG+GG | 400 (62.9%) | 529 (66.1%) | 1.152 (0.927-1.432) | p=0.203 |
| **rs2929973** |  |  |  |  |
| TT | 270 (42.5%) | 349 (43.6%) | 1.00 |  |
| TG | 297 (46.7%) | 348 (43.5%) | 0.906 (0.726-1.132) | p=0.386 |
| GG | 69 (10.8%) | 103 (12.9%) | 1.155 (0.819-1.629) | p=0.412 |
| TG+GG | 366 (57.5%) | 451 (56.4%) | 0.953 (0.772-1.176) | p=0.656 |

The odds ratio (OR) with their 95% confidence intervals were estimated by logistic regression models.

**Table B.** Clinical statuses and *WISP1* rs16893344 genotype frequencies in oral cancer among 193 non-betel quid chewers.

| Variable | ***WISP1* rs16893344 (non-betel quid chewers)** | | | |
| --- | --- | --- | --- | --- |
|  | CC  (n=144) n (%) | CT+TT (n=49) n (%) | OR (95% CI) | p value |
| **Clinical Stage** |  |  |  |  |
| Stage I/II | 71 (49.3%) | 24 (49.0%) | 1.00 | p=0.969 |
| Stage III/IV | 73 (50.7%) | 25 (51.0%) | 1.013 (0.530-1.938) |  |
| **Tumor size** |  |  |  |  |
| <T2 | 91 (63.2%) | 31 (63.3%) | 1.00 | p=0.993 |
| > T2 | 53 (36.8%) | 18 (36.7%) | 0.997 (0.509-1.953) |  |
| **Lymph node metastasis** |  |  |  |  |
| No | 95 (66.0%) | 30 (61.2%) | 1.00 | p=0.548 |
| Yes | 49 (34.0%) | 19 (38.8%) | 1.228 (0.628-2.400) |  |
| **Distant metastasis** |  |  |  |  |
| No | 143 (99.3%) | 48 (98.0%) | 1.00 | p=0.421 |
| Yes | 1 (0.7%) | 1 (2.0%) | 2.979 (0.183-48.554) |  |
| **Cell differentiation** |  |  |  |  |
| well | 48 (12.5%) | 4 (8.2%) | 1.00 | p=0.409 |
| Moderate/poor | 126 (87.5%) | 45 (91.8%) | 1.607 (0.516-5.003) |  |

**Table C.** Clinical statuses and *WISP1* rs2929970 genotype frequencies in oral cancer among 800 smoker.

| Variable | ***WISP1* rs2929970** | | | |
| --- | --- | --- | --- | --- |
|  | AA  (n=271) n (%) | AG+GG (n=529) n (%) | OR (95% CI) | p value |
| **Clinical Stage** |  |  |  |  |
| Stage I/II | 130 (48.0%) | 261 (49.3%) | 1.00 | p=0.714 |
| Stage III/IV | 141 (52.0%) | 268 (50.7%) | 0.947 (0.706-1.269) |  |
| **Tumor size** |  |  |  |  |
| <T2 | 155 (57.2%) | 303 (57.3%) | 1.00 | p=0.982 |
| > T2 | 116 (42.8%) | 226 (42.7%) | 0.997 (0.741-1.340) |  |
| **Lymph node metastasis** |  |  |  |  |
| No | 178 (65.7%) | 363 (68.6%) | 1.00 | p=0.401 |
| Yes | 93 (34.3%) | 166 (31.4%) | 0.875 (0.641-1.194) |  |
| **Distant metastasis** |  |  |  |  |
| No | 267 (98.5%) | 523 (98.9%) | 1.00 | p=0.680 |
| Yes | 4 (1.5%) | 6 (1.1%) | 0.766 (0.214-2.737) |  |
| **Cell differentiation** |  |  |  |  |
| well | 37 (13.7%) | 81 (15.3%) | 1.00 | p=0.531 |
| Moderate/poor | 234 (86.3%) | 448 (84.7%) | 0.875 (0.575-1.331) |  |
